# Dynamic Changes in Macrophage Infiltration and Polarization During Oral Carcinogenesis and Tumor Progression

**DOI:** 10.3390/biomedicines14102169

**Published:** 2026-09-24

**Authors:** Takuma Arashima, Kiyofumi Takabatake, Yulu Zhao, Ziyu Liu, Zixiao Li, Hotaka Kawai, Htoo Shwe Eain, Zin Zin Min, Keisuke Nakano, Hitoshi Nagatsuka

**Affiliations:** Department of Oral Pathology and Medicine, Graduate School of Medicine, Dentistry and Pharmaceutical Sciences, Okayama University, Okayama 700-8525, Japan; p5fk8v9b@s.okayama-u.ac.jp (T.A.); pm593ijm@s.okayama-u.ac.jp (Y.Z.); p96q9d1f@s.okayama-u.ac.jp (Z.L.); puai9btm@s.okayama-u.ac.jp (Z.L.); de18018@s.okayama-u.ac.jp (H.K.); pmp61kpp@s.okayama-u.ac.jp (H.S.E.); pobk1heq@s.okayama-u.ac.jp (Z.Z.M.); pir19btp@okayama-u.ac.jp (K.N.)

**Keywords:** oral squamous cell carcinoma (OSCC), oral epithelial dysplasia (OED), tumor-associated macrophages (TAMs), c-c motif chemokine ligand 2 (CCL2), tumor microenvironment (TME)

## Abstract

**Background:** Oral epithelial dysplasia (OED) is a precursor lesion to oral squamous cell carcinoma (OSCC). However, the dynamic changes in macrophage infiltration and polarization during oral carcinogenesis remain unclear. This study investigated macrophage infiltration and polarization during the progression from OED to OSCC, with particular emphasis on changes in the tumor microenvironment (TME). **Methods:** Human OED and OSCC specimens were immunohistochemically examined using macrophage markers representing the M0, M1, and M2 phenotypes. In addition, an indirect co-culture model using the OSCC cell lines HSC-2 and HSC-3 and RAW264.7 macrophages was established to evaluate macrophage migration and polarization in vitro. **Results:** Both M1- and M2-like macrophage populations increased with the histological progression of OED. M1-like macrophages predominated throughout the dysplastic stage, whereas M2-like macrophages became the predominant population after early stromal invasion. In OSCC, well-differentiated squamous cell carcinoma (SCC) was associated with greater macrophage infiltration, whereas poorly differentiated SCC was associated with a more M2-like macrophage polarization. **Conclusions:** The immune microenvironment begins to remodel during the precancerous stage of oral carcinogenesis. Macrophage recruitment and polarization differ according to the histological differentiation of OSCC, highlighting the importance of considering intratumoral histological heterogeneity when evaluating macrophage dynamics within the TME.

## 1. Introduction

Oral squamous cell carcinoma (OSCC) is the most common type of carcinoma in the oral cavity, accounting for approximately 90% of oral malignant neoplasms [1]. OSCC is composed of neoplastic epithelial cells (tumor parenchyma) and the surrounding stromal components. These two components interact dynamically and reciprocally, forming a complex tumor microenvironment (TME) that plays a pivotal role in tumor progression, invasion, metastasis, and therapeutic response [2]. The TME comprises various cellular and non-cellular components, including fibroblasts, macrophages, other immune cells, blood vessels, and the extracellular matrix (ECM). Among these components, macrophages are one of the most abundant and functionally important immune cell populations. Macrophages that infiltrate tumor tissues are referred to as tumor-associated macrophages (TAMs). Extensive evidence has demonstrated that TAMs contribute to multiple aspects of tumor progression, including cancer cell proliferation, invasion, angiogenesis, immune evasion, and lymph node metastasis [3,4]. Macrophages are broadly classified into two functional phenotypes: M1 macrophages, characterized by the expression of CD11c, CD86, CD80, and inducible nitric oxide synthase (iNOS), and M2 macrophages, which express CD163, CD204, and CD206 [5,6]. M1 macrophages exert antitumor activity through the production of pro-inflammatory cytokines and cytotoxic mediators, whereas M2 macrophages promote tumor progression by enhancing immunosuppression, angiogenesis, and ECM remodeling [7,8,9]. TAMs predominantly exhibit an M2-like phenotype and commonly express CD163 and CD206 [10]. Among the various chemokines involved in TAM recruitment and polarization, C-C motif chemokine ligand 2 (CCL2) plays a pivotal role in promoting polarization toward the M2-like phenotype within the TME [8].

OSCC exhibits varying degrees of histological differentiation and is generally classified as well, moderately, or poorly differentiated. A previous study reported that poorly differentiated tumors are associated with increased infiltration of TAMs [11]. However, surgical specimens often contain regions with different histological grades of differentiation within the same tumor. Despite this histological heterogeneity, few studies have comprehensively investigated the pathological relationship between the degree of tumor differentiation and the distribution of TAMs within individual OSCC lesions.

Oral epithelial dysplasia (OED) is one of the oral potentially malignant disorders that represent precursor lesions to OSCC [12]. Increasing evidence indicates that alterations in the microenvironment occur during the early stages of oral carcinogenesis, even before the development of invasive carcinoma [13]. Although the role of TAMs has been extensively investigated in established OSCC, relatively few studies have examined their involvement during the earlier stages of oral carcinogenesis. Current evidence suggests that macrophage infiltration, particularly that of CD163-positive macrophages, increases with the severity of OED and may be associated with an increased risk of malignant transformation [14]. However, these observations have largely been based on analyses of OED alone, and it remains unclear how the macrophage microenvironment changes during the transition from OED to invasive OSCC. In particular, whether macrophage infiltration and polarization undergo a distinct shift at the acquisition of invasive potential, and how this shift relates to the macrophage landscape of established OSCC, remain poorly understood. Therefore, investigating OED alone would not fully address the dynamic relationship between macrophages and oral carcinogenesis. A comparative analysis spanning OED, early invasive OSCC, and infiltration and polarization change dynamically along the continuum of oral carcinogenesis and tumor progression.

Therefore, in this study, we investigated macrophage dynamics changes in macrophage infiltration and polarization across the continuum of oral carcinogenesis, from OED to invasive OSCC. Using human tongue tissue specimens, we specifically examined the infiltration patterns and polarization state of macrophages across sequential pathological stages, including OED, early invasive OSCC, and established OSCC, to determine whether the macrophage microenvironment changes during the transition from dysplasia to invasive carcinoma. Furthermore, to investigate the influence of tumor differentiation on macrophage behavior, we performed in vitro experiments using the well-differentiated OSCC cell line HSC-2, the poorly differentiated OSCC cell line HSC-3, and the murine macrophage cell line RAW264.7 to evaluate macrophage infiltration, polarization, and migratory activity.

## 2. Materials and Methods

### 2.1. Carcinogenesis Experiment

All animal experiments were conducted in accordance with the guidelines and regulations of the Animal Care and Use Committee of Okayama University and were approved by the committee (Approval No. 2025262).

A total of 43 female C57BL/6J mice were used in this study. Female C57BL/6J mice were selected because this strain and sex have been widely used in established 4-nitroquinoline 1-oxide (4NQO)-induced oral carcinogenesis models [15,16]. 4NQO (FUJIFILM Wako Pure Chemical Corporation, Tokyo, Japan) was dissolved in ethanol and diluted to a final concentration of 200 μg/mL immediately before use. Of the 43 mice, 38 received topical application of 4NQO to the right lateral border of the tongue every other day for 16 weeks, whereas five mice received topical physiological saline using the same procedure and served as normal controls. All animals were euthanized simultaneously at the end of the 16-week experimental period, and oral tissues were collected for histopathological examination.

Histopathological examination of the 4NQO-treated animals identified six mice with oral epithelial dysplasia (OED) and two mice with squamous cell carcinoma (SCC). Tissues from these animals were used for subsequent analyses. Thus, the normal control, OED, and SCC groups comprised 5, 6, and 2 mice, respectively.

### 2.2. Human Tissue Specimen Selection

This retrospective study included human tissue specimens that were histologically diagnosed in the Department of Oral Pathology, Okayama University, between 2012 and 2019. A total of 16 cases of low-grade dysplasia, 16 cases of high-grade dysplasia, and 8 cases of early invasive OSCC (early invasive squamous cell carcinoma [SCC]) were selected from biopsy and surgical resection specimens. Early invasive SCC was defined as a lesion with a depth of invasion < 3 mm. Regarding OED, epithelial dysplasia was diagnosed according to the WHO diagnostic criteria. Low-grade and high-grade dysplasia were classified based on the number of architectural and cytological atypical features, with four architectural abnormalities and five cytological abnormalities used as the respective cutoff values.

In addition, 32 primary OSCC cases classified as pT1 or pT2, with or without recurrence or secondary lymph node metastasis, were included. The cohort comprised 16 well-differentiated SCCs and 16 moderately or poorly differentiated SCCs. Only primary tumor specimens were analyzed in this study. Patient with lymph node metastasis at the time of surgery were excluded to investigate the impact on secondary metastasis. Cases of recurrent OSCC and patients who had received preoperative chemotherapy and/or radiotherapy were excluded from the analysis. Regarding OSCC, tumor staging was performed according to the TNM classification of malignant tumors established by the Union for International Cancer Control (UICC).

To minimize observer bias, all specimens were independently evaluated by two oral pathologists. In cases of disagreement, a third oral pathologist independently reviewed the specimens, and a consensus diagnosis was reached through discussion among the three pathologists.

### 2.3. Hematoxylin and Eosin (H&E) Staining

Tissue samples were fixed in 4% paraformaldehyde (PFA; Nacalai Tesque, Kyoto, Japan), embedded in paraffin, sectioned at a thickness of 4 μm, and stained with H&E.

### 2.4. Immunohistochemistry Staining

Immunohistochemical staining was performed on 3 μm thick formalin-fixed, paraffin-embedded (FFPE) tissue sections. The FFPE tissue sections were deparaffinized in xylene, rehydrated through a graded ethanol series, and endogenous peroxidase activity was blocked with 0.3% hydrogen peroxide in methanol for 30 min at room temperature.

Antigen retrieval was performed either by heat-induced epitope retrieval using a microwave oven or by enzymatic digestion with trypsin, depending on the antibody used. After blocking with 10% normal serum for 15 min at room temperature, the sections were incubated overnight at 4 °C with the following primary antibodies: anti-CD68 (rabbit polyclonal, 1:100, cat. no. sc-9139, Santa Cruz Biotechnology, Dallas, TX, USA), anti-F4/80 (rat monoclonal, 1:10, cat. no. MCA497, Bio-Rad Laboratories, Hercules, CA, USA), anti-CD11c (mouse monoclonal, 1:500, cat. no. 60258-1-Ig, Proteintech, Rosemont, IL, USA), anti-iNOS (rabbit polyclonal, 1:200, cat. no. 18985-1-AP, Proteintech), anti-CD163 (rabbit polyclonal, 1:200, cat. no. ab182422, Abcam, Cambridge, UK), and anti-CCL2 (rabbit polyclonal, 1:50, cat. no. ab315478, Abcam). CD68 and F4/80 were used as markers of macrophages, CD11c and iNOS as markers of M1-like macrophages, CD163 as a marker of M2-like macrophages, and CCL2 was evaluated because of its role in macrophage recruitment.

Immunoreactivity was visualized using the avidin–biotin complex (ABC) method (Rabbit/Mouse/Rat ABC Kit; Vector Laboratories, Newark, CA, USA) according to the manufacturer’s instructions. Immunoreactive signals were developed with 3,3′-diaminobenzidine tetrahydrochloride, and the sections were counterstained with hematoxylin.

For quantitative analysis, five representative hotspot fields were selected from each specimen. Images were captured at ×40 magnification for CD68, F4/80, CD11c, iNOS, and CD163 and at ×20 magnification for CCL2 using a BX51 light microscope (Olympus Corporation, Tokyo, Japan). The numbers of CD68-, F4/80-, CD11c-, iNOS-, CD163-, and CCL2-positive cells were quantified using ImageJ software (Version 1.54t) (National Institutes of Health, Bethesda, MD, USA).

### 2.5. Cell Lines and Cell Culture

The well-differentiated human OSCC cell line HSC-2 (JCRB0622) and the poorly differentiated human OSCC cell line HSC-3 (JCRB0623) were obtained from the Japanese Collection of Research Bioresources (Osaka, Japan). The murine macrophage cell line RAW264.7 (RCB0535) was obtained from the RIKEN Bioresource Center Cell Bank (Tsukuba, Ibaraki, Japan).

HSC-2, HSC-3, and RAW264.7 cells were cultured in α-minimum essential medium (α-MEM; Thermo Fisher Scientific, Waltham, MA, USA) supplemented with 10% fetal bovine serum (FBS; Biowest, Nuaillé, France) and 100 U/mL penicillin–streptomycin (Thermo Fisher Scientific). The cells were maintained at 37 °C in a humidified incubator containing 5% CO2 and 95% air.

Although HSC-2 and HSC-3 cells are of human origin, whereas RAW264.7 cells are derived from mice, this heterologous cell culture system was considered appropriate for the present study because it is widely used to investigate the interactions between human oral squamous cell carcinoma cells and macrophage/osteoclast-lineage cells [17,18,19].

### 2.6. Migration Assay

RAW264.7, HSC-2, and HSC-3 cells were cultured until they reached approximately 90% confluence and were then harvested for the migration assay. Macrophage migration was evaluated using 24-well Transwell inserts with 8.0 μm transparent polyethylene terephthalate (PET) membranes (Corning, Corning, NY, USA).

Briefly, 2 × 10^4^ RAW264.7 cells suspended in 500 μL of serum-free α-MEM were seeded into the upper chamber. HSC-2 or HSC-3 cells (5 × 10^4^ cells/well) suspended in 500 μL of α-MEM supplemented with 10% FBS were seeded into the lower chamber as chemoattractant cells. After incubation for 24 h at 37 °C in a humidified atmosphere containing 5% CO_2_, the nonmigrated cells remaining on the upper surface of the membrane were gently removed with a cotton swab. The migrated cells on the lower surface of the membrane were fixed and stained using a Diff-Quick Giemsa staining kit (Sysmex Corporation, Kobe, Japan). The membranes were then excised, mounted on glass slides, and examined under a light microscope.

Five randomly selected fields per membrane were photographed at ×20 magnification, and the number of migrated cells was quantified. Each experiment was performed independently in triplicate.

### 2.7. Indirect Co-Culture (Macrophage Polarization Analysis) and Double-Immunofluorescence Staining

RAW264.7, HSC-2, and HSC-3 cells were cultured until they reached approximately 90% confluence and were then harvested for the indirect co-culture experiments. RAW264.7 cells were seeded into the lower wells of a 24-well plate at a density of 7 × 10^4^ cells/well in 500 μL of α-MEM supplemented with 10% FBS. HSC-2 or HSC-3 cells were seeded into Transwell inserts with 8.0 μm pore-size PET membranes (Corning, Corning, NY, USA) at a density of 5 × 10^4^ cells/well in 500 μL of α-MEM supplemented with 10% FBS. The cells were indirectly co-cultured for 6 days.

On day 3, the culture medium in the lower chamber was replaced with fresh α-MEM supplemented with 10% FBS after the RAW264.7 cells were washed with Tris-buffered saline (TBS). Simultaneously, the Transwell inserts containing HSC-2 or HSC-3 cells were replaced with newly prepared inserts seeded with the corresponding tumor cells. On day 6, the RAW264.7 cells were subjected to immunofluorescence staining.

After washing with TBS, RAW264.7 cells were fixed with 4% PFA for 15 min at room temperature and blocked with a blocking solution for 20 min. The cells were then incubated for 60 min with the following primary antibodies: anti-F4/80 (rat monoclonal, 1:10, cat. no. MCA497, Bio-Rad Laboratories, Hercules, CA, USA), anti-iNOS (rabbit polyclonal, 1:200, cat. no. 18985-1-AP, Proteintech), and anti-CD163 (rabbit polyclonal, 1:200, cat. no. ab182422, Abcam). F4/80 was co-stained with either iNOS or CD163 by simultaneous incubation with the corresponding primary antibodies.

After three washes with TBS, the cells were incubated for 80 min at room temperature in the dark with the following secondary antibodies: Alexa Fluor 488-conjugated goat anti-rat IgG (1:200; Thermo Fisher Scientific, Waltham, MA, USA) and Alexa Fluor 568-conjugated goat anti-rabbit IgG (1:200; Thermo Fisher Scientific). The cells were subsequently washed three times with TBS, rinsed twice with distilled water, and counterstained with 4′,6-diamidino-2-phenylindole (DAPI) (SouthernBiotech, Birmingham, AL, USA).

Fluorescence images were acquired using a BZ-X700 All-in-One fluorescence microscope (Keyence, Osaka, Japan). Five representative hotspot fields were photographed at ×20 magnification. F4/80-, iNOS-, and CD163-positive cells were counted. In addition, the proportions of iNOS-positive/F4/80-positive and CD163-positive/F4/80-positive cells were calculated. Each experiment was performed independently in triplicate.

### 2.8. Statistical Analysis

Statistical analyses were performed using GraphPad Prism 11 (GraphPad Software Inc., San Diego, CA, USA).

All in vitro experiments were performed independently at least three times, and the data are presented as the mean ± standard deviation (SD).

Comparisons between two groups were performed using the unpaired Student’s *t*-test. Comparisons among three or more groups were performed using one-way analysis of variance, followed by Tukey’s multiple-comparison test. A *p* value < 0.05 was considered statistically significant.

## 3. Results

### 3.1. Macrophage Infiltration During Oral Carcinogenesis in the Mouse Model

Representative H&E and immunohistochemical staining for F4/80, iNOS, and CD163 are shown in Figure 1A–L. Compared with human tissues, the mouse oral carcinogenesis model exhibited a thinner lamina propria throughout oral carcinogenesis (Figure 1A,E,I). Furthermore, OED in mice exhibited milder cytological and architectural atypia than that typically observed in human OED.

F4/80-positive and iNOS-positive cells exhibited a predominantly round morphology and were distributed adjacent to the dysplastic epithelium. In contrast, CD163-positive cells were relatively larger and exhibited a dendritic morphology.

Quantitative analysis demonstrated that the number of F4/80-positive cells increased significantly from normal epithelium to OED (*p* = 0.0013), followed by a significant decrease from OED to SCC (*p* = 0.050). Although the number of iNOS-positive cells showed an increasing trend during oral carcinogenesis, the differences did not reach statistical significance. The number of CD163-positive cells increased significantly from normal epithelium to OED (*p* = 0.0002), whereas no significant difference was observed between OED and SCC (Figure 1M). Notably, CD163-positive cells were consistently more abundant than F4/80- or iNOS-positive cells throughout all stages of oral carcinogenesis.

### 3.2. Macrophage Infiltration in OED and Early Invasive SCC in Human Tissues

The clinical findings of OED and early invasive SCC cases are summarized in Table 1, and representative H&E and immunohistochemical staining for CD68, CD11c, and CD163 in low-grade dysplasia, high-grade dysplasia, and early invasive SCC are shown in Figure 2A–L.

In low-grade dysplasia, the basal and parabasal cells exhibited mild nuclear enlargement and cellular atypia compared with those in normal epithelium (Figure 2A). In high-grade dysplasia, cytological and architectural atypia were observed not only in the basal and parabasal cells but also in the spinous cell layer (Figure 2E). In early invasive SCC, the epithelial rete ridges became indistinct because of stromal invasion and were accompanied by prominent inflammatory cell infiltration in the adjacent connective tissue (Figure 2I).

Quantitative analysis demonstrated that the number of CD163-positive cells increased progressively with histological grade, with a marked increase from low-grade dysplasia to high-grade dysplasia (*p* = 0.00010). Similarly, the number of CD68-positive cells increased significantly during disease progression. CD11c-positive cells also increased significantly from low-grade dysplasia to high-grade dysplasia (*p* < 0.0001); however, their number decreased significantly in early invasive SCC compared with high-grade dysplasia (*p* = 0.027) (Figure 2M).

The temporal changes in the mean numbers of macrophages expressing each marker across the histological stages are shown in Figure 2N. The mean numbers of CD68-positive and CD163-positive cells increased progressively throughout disease progression. In contrast, the number of CD11c-positive cells decreased during the transition from high-grade dysplasia to early invasive SCC.

Representative immunohistochemical staining for CCL2 is shown in Figure 2O. In OED, CCL2-positive cells were predominantly localized to the basal and spinous cell layers. In contrast, in early invasive SCC, CCL2-positive cells were distributed throughout the entire epithelial layer. Quantitative analysis revealed that the number of CCL2-positive cells tended to increase from low-grade dysplasia to high-grade dysplasia; however, the difference was not statistically significant. In contrast, a significant increase was observed from high-grade dysplasia to early invasive SCC (*p* = 0.0094) (Figure 2P).

### 3.3. Macrophage Infiltration and Polarization in OSCC According to Tumor Stage and Clinical Outcome

The clinical findings of OSCC cases are summarized in Table 2. In the T1 OSCC group, the age of patients was 27–83, and it was 28–79 in the T2 OSCC group. Representative H&E and immunohistochemical staining for CD68, CD11c, and CD163 in pT1 and pT2 cases are shown in Figure 3A–P. Compared with OED, both pT1 and pT2 cases exhibited more prominent inflammatory cell infiltration within the tumor stroma, accompanied by an increased number of macrophages expressing each marker.

Quantitative analysis demonstrated no significant differences in the numbers of CD68-positive or CD11c-positive cells between pT1 and pT2 cases (Figure 3Q,R). However, the number of CD163-positive cells was significantly higher in pT2 cases than in pT1 cases (*p* = 0.0019) (Figure 3S).

In pT1 cases, no significant difference was observed between the numbers of CD68-positive and CD163-positive cells (Figure 3T). However, in pT2 cases, CD163-positive macrophages were significantly more abundant than CD68-positive macrophages (*p* < 0.0001) (Figure 3U).

No significant difference was observed in the CD11c/CD68 ratio between pT1 and pT2 cases (Figure 3V). In contrast, the CD163/CD68 ratio was significantly higher in pT2 cases than in pT1 cases (*p* = 0.0056) (Figure 3W), indicating that the proportion of CD163-positive macrophages increased with tumor stage.

Furthermore, no significant differences were observed in the numbers of CD68-, CD11c-, or CD163-positive cells between patients with and without recurrence or secondary lymph node metastasis (Figure 3X).

### 3.4. Macrophage Infiltration and Polarization in Relation to Histological Differentiation

When OSCC cases were classified according to histological differentiation, macrophage infiltration differed depending on the macrophage marker (Figure 4A–C). Compared with well-differentiated SCC, the number of CD68-positive cells was significantly higher in moderately or poorly differentiated SCC (*p* < 0.0001). In contrast, the number of CD11c-positive cells was significantly higher in well-differentiated SCC (*p* = 0.046), whereas no significant difference was observed in the number of CD163-positive cells between the two groups (*p* = 0.98).

Histological differentiation in OSCC is conventionally classified according to the predominant pattern of differentiation within the tumor. However, histopathological examination revealed substantial intratumoral heterogeneity, with different degrees of differentiation frequently coexisting within individual tumors (Figure 4D–H). Therefore, to evaluate macrophage infiltration while accounting for this histological heterogeneity, regional comparisons were performed between well-differentiated and poorly differentiated regions within the same tumor. To further evaluate macrophage infiltration according to local histological differentiation, five OSCC cases exhibiting distinct intratumoral heterogeneity were selected for regional analysis. In contrast to the comparison based on the overall histological differentiation of the tumors, all macrophage markers exhibited a consistent pattern within individual tumors. The numbers of CD68-, CD11c-, and CD163-positive cells were significantly higher in well-differentiated regions than in poorly differentiated regions (CD68, *p* = 0.0026; CD11c, *p* = 0.0069; CD163, *p* = 0.0003) (Figure 4I–K).

### 3.5. Macrophage Infiltration and Polarization in Vitro

The migration assay demonstrated that significantly more RAW264.7 cells migrated toward HSC-2 cells than toward HSC-3 cells, indicating that HSC-2 cells possess a greater macrophage chemoattractant capacity (Figure 5A,B).

Moreover, the co-culture experiments demonstrated distinct effects of the two OSCC cell lines on macrophage infiltration and polarization (Figure 5C). The numbers of F4/80-positive and iNOS-positive cells were significantly higher in the RAW264.7 + HSC-2 group than in the RAW264.7 + HSC-3 group (both *p* < 0.0001) (Figure 5D,E). Although the number of CD163-positive cells showed a decreasing trend in the order of the RAW264.7 + HSC-2 group, the RAW264.7 + HSC-3 group, and the RAW264.7-only group, the differences were not statistically significant (Figure 5F).

When macrophage polarization was evaluated as the proportion of marker-positive cells among F4/80-positive macrophages, no significant difference was observed in the iNOS/F4/80 ratio between the RAW264.7 + HSC-2 and RAW264.7 + HSC-3 groups, although the ratio tended to be higher in the RAW264.7 + HSC-3 group (Figure 5G). In contrast, the CD163/F4/80 ratio was significantly higher in the RAW264.7 + HSC-3 group than in the RAW264.7 + HSC-2 group (*p* < 0.0001) (Figure 5H).

## 4. Discussion

In the present study, the number of CD163-positive macrophages gradually increased from low-grade dysplasia to high-grade dysplasia, indicating that macrophage polarization toward the M2 phenotype begins before malignant transformation. Previous studies have similarly reported increased infiltration of M2 macrophages in OED and its association with malignant transformation [14,20,21]. Interestingly, M1 macrophages remained the predominant population throughout the dysplastic stages, suggesting that the inflammatory microenvironment of OED is characterized by active immune surveillance despite the progressive accumulation of M2-like macrophages. Because CCL2 expression did not differ significantly between low-grade and high-grade dysplasia, factors other than the CCL2–CCR2 axis may contribute to macrophage recruitment during early oral carcinogenesis [22]. Collectively, these findings indicate that remodeling of the immune microenvironment begins during the precancerous stage, before overt invasion occurs.

The findings obtained from the 4NQO-induced mouse model differed from those observed in human tissues. Although M2-like macrophages predominated in murine lesions, the total number of infiltrating macrophages did not increase with disease progression. Similar observations were reported by Miki et al. [23]. This discrepancy may reflect intrinsic differences between experimental carcinogenesis models and human OSCC, including anatomical differences in the oral mucosa, species-specific immune responses, and the relatively simplified TME in murine lesions [23].

One of the most important findings of the present study was the dynamic shift in macrophage polarization during oral carcinogenesis. M1-like macrophages were the predominant population throughout the dysplastic stages, whereas M2-like macrophages became predominant following early stromal invasion. These findings suggest that the shift in macrophage polarization from an M1- to an M2-dominant phenotype represents a critical immunological event accompanying the transition from OED to invasive OSCC, rather than a gradual process occurring throughout dysplasia. Such a shift may provide a useful adjunctive indicator for histopathological assessment, particularly in borderline lesions in which stromal invasion is difficult to determine.

Another notable finding of this study was the influence of intratumoral histological heterogeneity on macrophage infiltration. Histological grading of OSCC is conventionally determined according to the predominant pattern of differentiation within the tumor. However, individual tumors frequently contain well-, moderately, and poorly differentiated components. When the cases were analyzed according to the overall histological grade, different macrophage markers exhibited inconsistent patterns. In contrast, regional analysis within the same tumors demonstrated that all macrophage markers were more abundant in well-differentiated regions. These observations indicate that the local histological architecture may have a greater influence on macrophage recruitment than the overall histological grade assigned to the tumor.

Although TAMs have been reported to promote lymphangiogenesis and lymph node metastasis in OSCC [3], their role in predicting postoperative recurrence remains controversial. In the present study, macrophage infiltration was not significantly associated with either tumor recurrence or secondary lymph node metastasis. Similar findings were reported by Kubota et al., who found no correlation between CD163- or CD204-positive macrophages and recurrence [24]. These observations suggest that the biological functions of TAMs may be more closely related to the establishment of the primary TME than to postoperative disease progression. Therefore, additional factors within the TME are likely to influence recurrence and secondary lymph node metastasis.

The in vitro findings further supported the histological observations. HSC-2 cells induced significantly greater macrophage migration than HSC-3 cells, whereas HSC-3 cells increased the proportion of CD163-positive macrophages among infiltrating macrophages. Consistent with these findings, regional analysis of human OSCC tissues demonstrated significantly greater infiltration of CD68-, CD11c-, and CD163-positive macrophages in well-differentiated regions than in poorly differentiated regions within the same tumor. Several previous studies have reported increased infiltration of CD163-positive macrophages in poorly differentiated OSCC [11,25], apparently contrasting with our findings. However, those studies classified tumors according to the predominant histological grade of each case, whereas the present study additionally evaluated macrophage infiltration within histologically distinct regions of the same tumor. Given the marked intratumoral heterogeneity of OSCC, regional analysis may more accurately reflect the local TME than conventional case-based histological grading. Collectively, these findings suggest that well-differentiated OSCC primarily promotes macrophage recruitment, whereas poorly differentiated OSCC preferentially promotes macrophage polarization toward the M2 phenotype. In other words, well-differentiated tumors recruit macrophages, whereas poorly differentiated tumors educate macrophages. These findings further suggest that macrophage recruitment and macrophage polarization represent distinct biological processes during OSCC progression.

One possible explanation for these findings is differential chemokine production according to tumor differentiation. CCL2, produced by both tumor cells and stromal cells, is a major regulator of macrophage recruitment through the CCR2 signaling pathway [8,26,27]. In addition, PDGF-BB-induced CCL2 expression has been reported to enhance macrophage recruitment through the CCL2–CCR2 axis [27]. Therefore, regional differences in CCL2 expression among histologically distinct tumor components may contribute to the differential recruitment and polarization of macrophages observed in the present study. Further mechanistic studies are warranted to clarify the molecular mechanisms underlying these observations.

A limitation of this study is that macrophage phenotypes were characterized using immunohistochemical markers rather than multiplex immunophenotyping or transcriptomic approaches. Although CD68/F4/80, CD11c/iNOS, and CD163 were used to characterize macrophage-associated phenotypes, these markers do not exclusively define functionally distinct M1 or M2 macrophage populations. In particular, CD11c may be expressed by other myeloid populations, and CD163 expression alone does not establish a functionally defined M2 state. Therefore, the M1-like and M2-like phenotypes described in this study should be interpreted as immunohistochemically defined phenotypic profiles rather than discrete functional macrophage subsets. Nevertheless, the consistent quantitative and spatial changes in these macrophage-associated phenotypes across the sequential stages of OED, early invasive OSCC, and established OSCC support the presence of dynamic macrophage remodeling during oral carcinogenesis and tumor progression. Further studies using multiplex immunostaining, single-cell transcriptomic analysis, and functional assays will be required to define the precise molecular and functional states of these macrophage populations.

Another limitation of this study is the use of a cross-species co-culture system consisting of human OSCC cell lines and murine RAW264.7 macrophages. Species differences in cytokine, chemokine, and receptor–ligand interactions may influence macrophage responses and therefore limit the direct extrapolation of the in vitro findings to human macrophages. Although the concordance between the in vitro findings and the observation in human tissue supports their biological relevance, the present experiments do not establish that the same tumor-derived signals operate in human macrophages. Future studies using human monocyte-derived macrophages or other human macrophage models will be necessary to further validate these findings.

## 5. Conclusions

This study demonstrated that remodeling of the immune microenvironment begins during the precancerous stage of oral carcinogenesis. Macrophage recruitment and polarization differed according to the histological differentiation of OSCC, highlighting the importance of considering intratumoral histological heterogeneity when evaluating the TME.

## Figures and Tables

**Figure 1:**
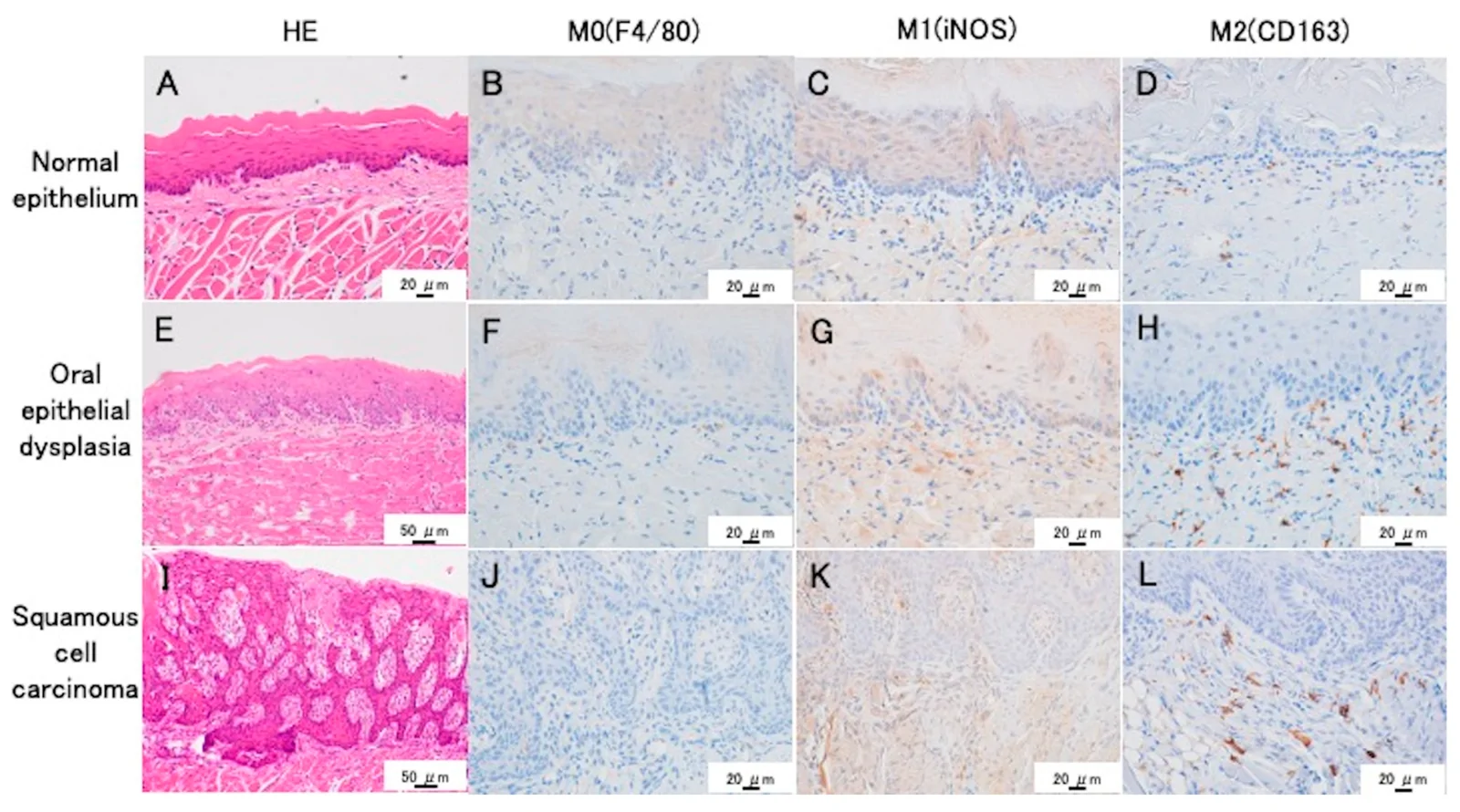

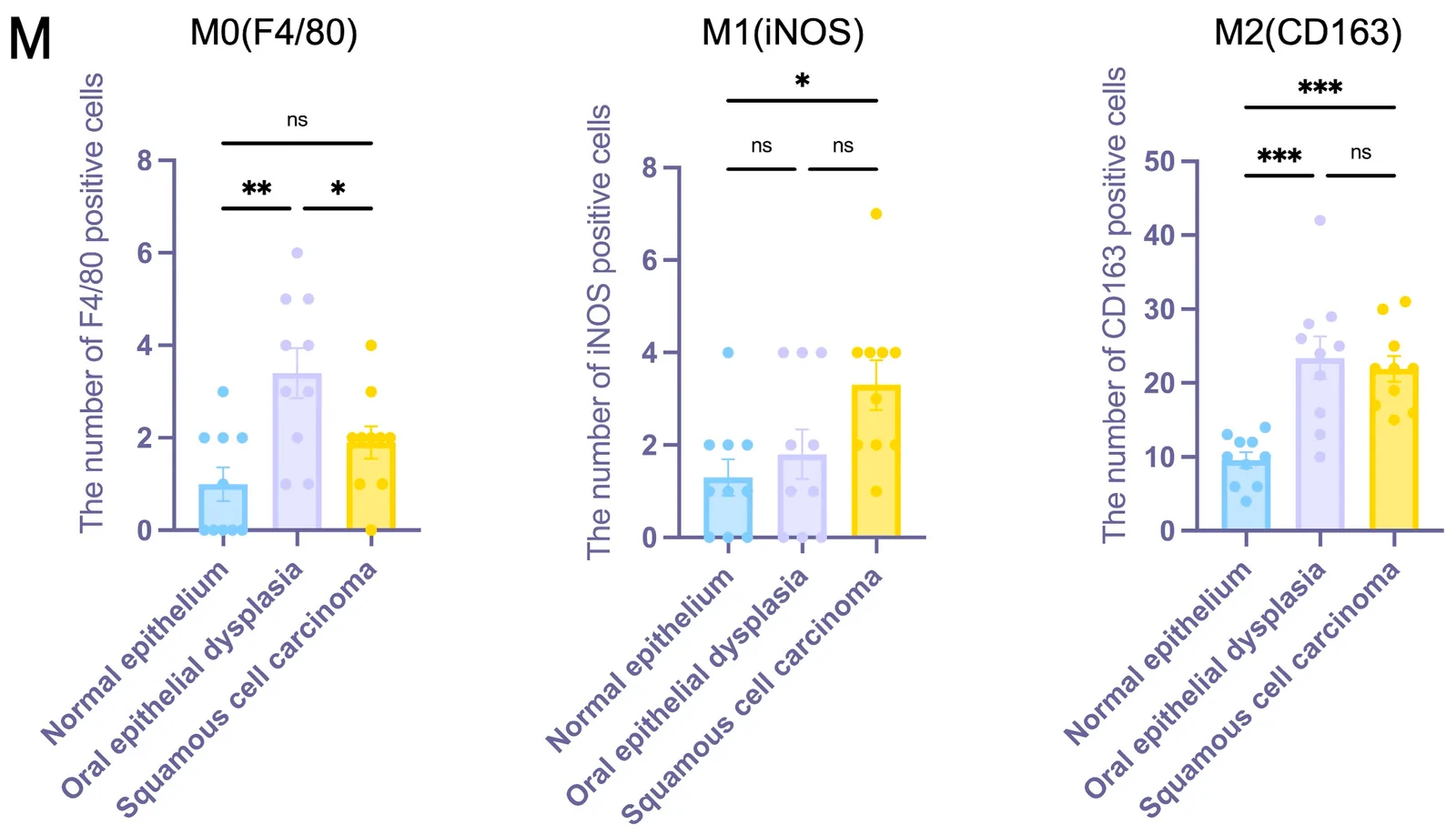
Macrophage infiltration during oral carcinogenesis in the mouse model. Representative photomicrographs of H&E staining (**A**,**E**,**I**) and immunohistochemical staining for F4/80 (**B**,**F**,**J**), iNOS (**C**,**G**,**K**), and CD163 (**D**,**H**,**L**). (**A**–**D**) Normal epithelium. (**E**–**H**) Oral epithelial dysplasia (OED). (**I**–**L**) Squamous cell carcinoma (SCC). (**M**) Quantification of F4/80-, iNOS-, and CD163-positive cells in each histological group. The number of F4/80-positive cells increased significantly in OED and subsequently decreased significantly in SCC (*p* < 0.01 and *p* < 0.05, respectively). No significant difference in the number of iNOS-positive cells was observed among the groups. The number of CD163-positive cells increased significantly from normal epithelium to OED (*p* < 0.001). Data are presented as the mean ± SD. ns, *p* > 0.05; * *p* < 0.05; ** *p* < 0.01; *** *p* < 0.001. Normal epithelium: n = 5, OED: n = 6, SCC: n = 2.

**Figure 2:**
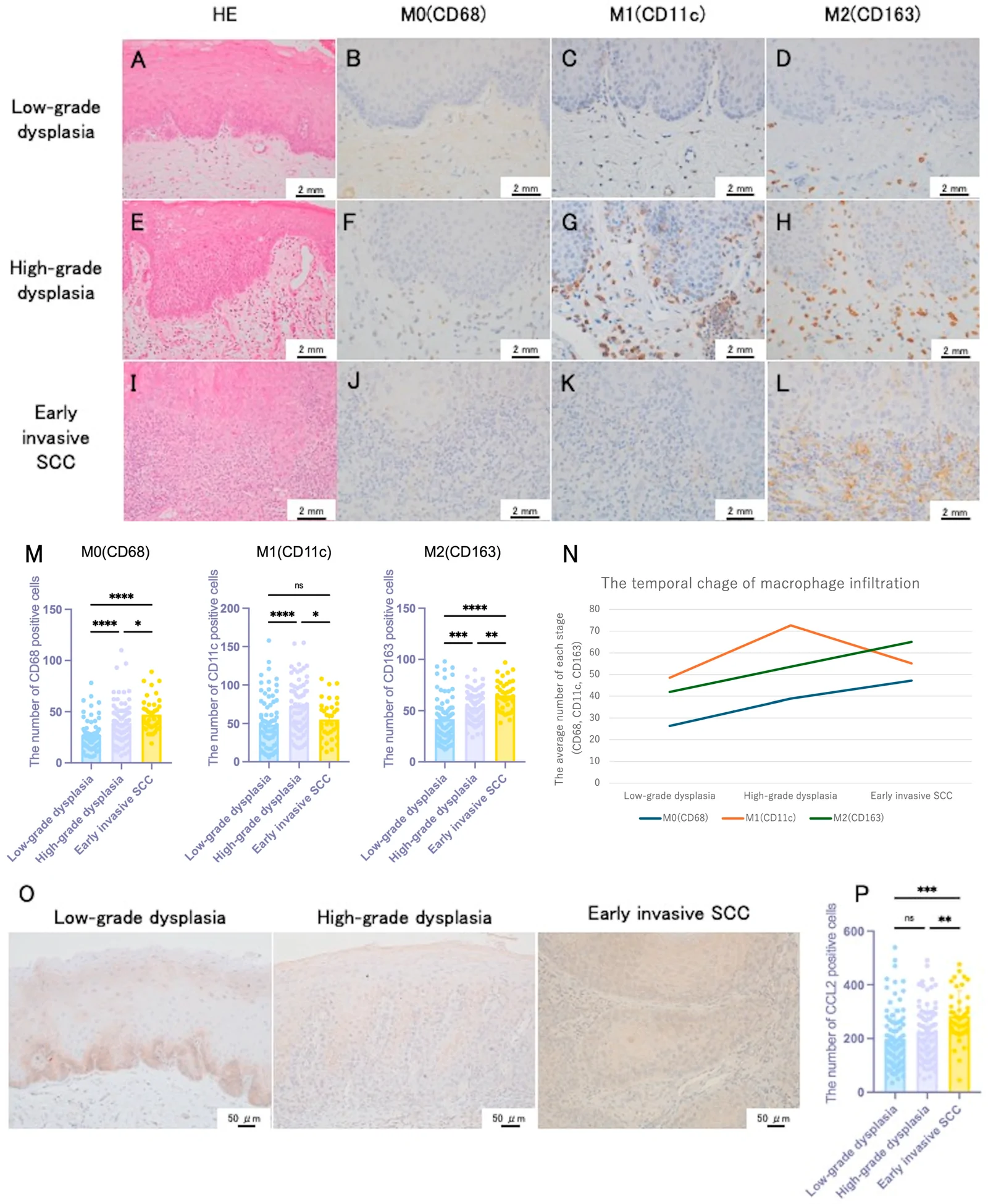
Macrophage infiltration and polarization during oral carcinogenesis in human tissues. Representative photomicrographs of H&E staining (**A**,**E**,**I**) and immunohistochemical staining for CD68 (**B**,**F**,**J**), CD11c (**C**,**G**,**K**), CD163 (**D**,**H**,**L**), and CCL2 (**O**). (**A**–**D**) Low-grade dysplasia. (**E**–**H**) High-grade dysplasia. (**I**–**L**) Early invasive SCC. (**M**) Quantification of CD68-, CD11c-, and CD163-positive cells in each histological grade. The numbers of CD68-positive and CD163-positive cells increased significantly with histological progression. In contrast, the number of CD11c-positive cells decreased significantly from high-grade dysplasia to early invasive SCC. (**N**) Temporal changes in the mean numbers of CD68-, CD11c-, and CD163-positive cells across the different histological grades. The mean numbers of CD68-positive and CD163-positive cells increased progressively, whereas the mean number of CD11c-positive cells decreased during the transition from high-grade dysplasia to early invasive SCC. (**O**) Representative immunohistochemical staining for CCL2. (**P**) Quantification of CCL2-positive cells. The number of CCL2-positive cells increased significantly from high-grade dysplasia to early invasive SCC. Data are presented as the mean ± standard deviation (SD). ns, *p* > 0.05; * *p* < 0.05; ** *p* < 0.01; *** *p* < 0.001; **** *p* < 0.0001. Low-grade dysplasia: n = 16, high-grade dysplasia: n = 16, early invasive SCC: n = 8.

**Figure 3:**
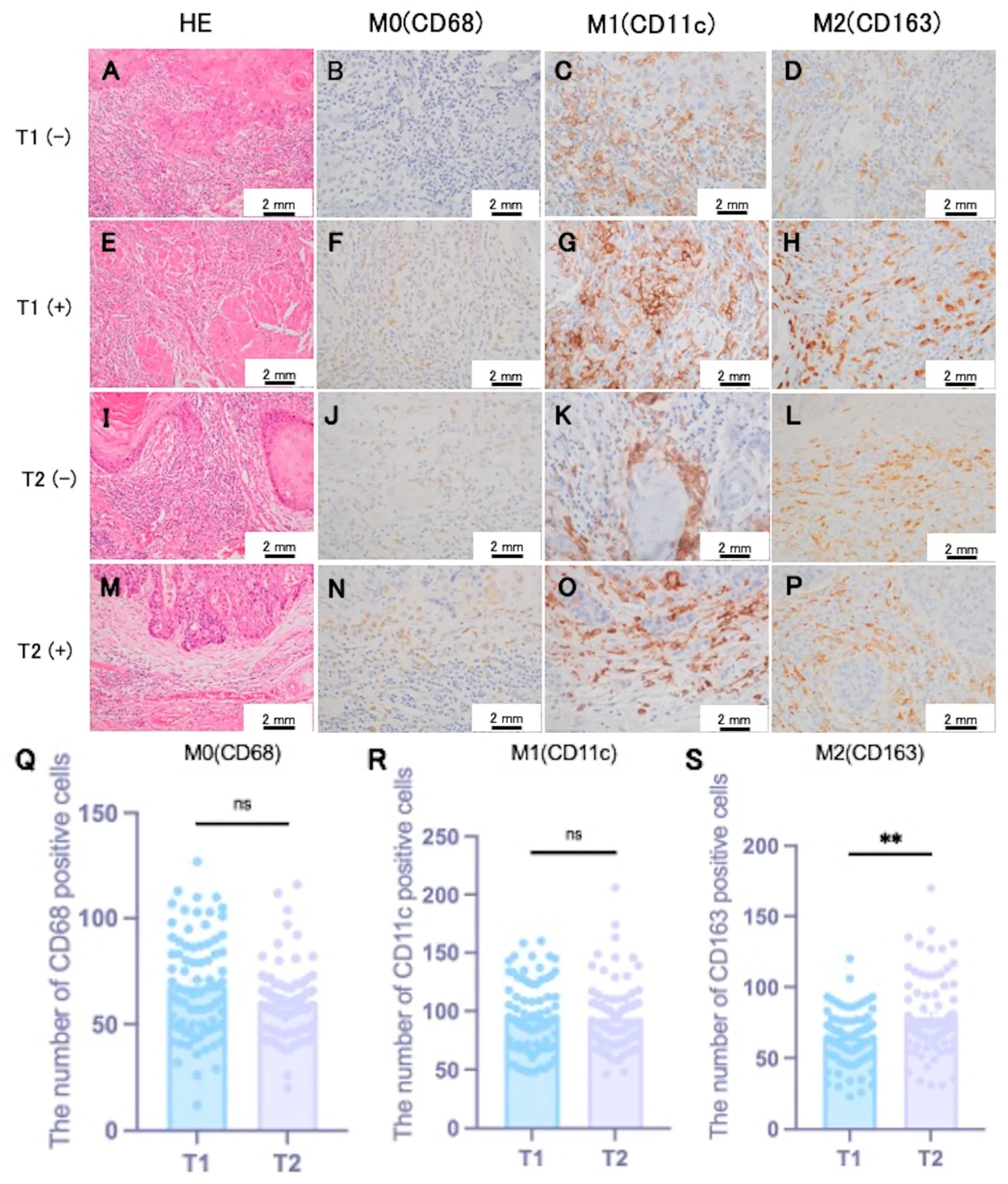

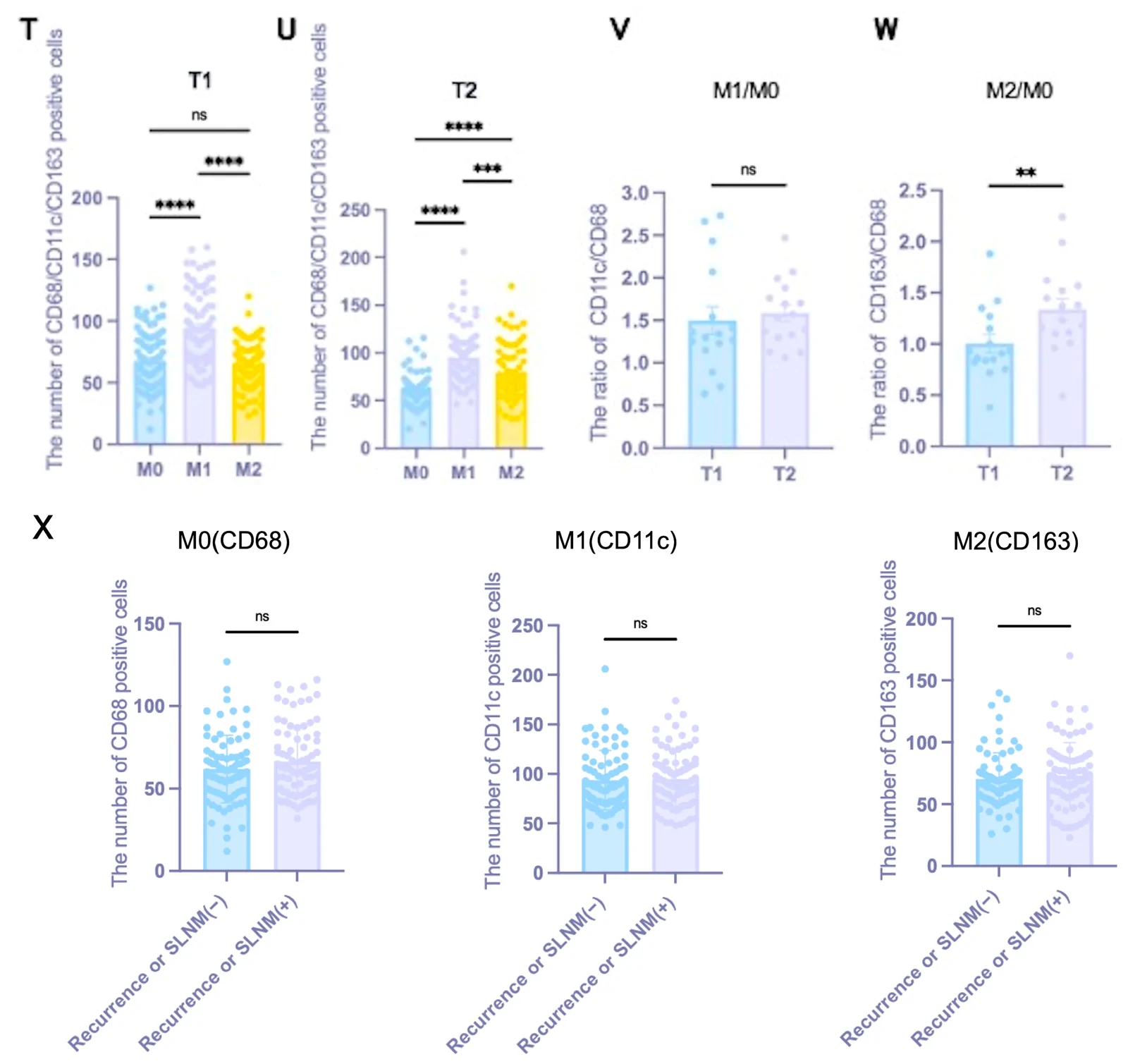
Macrophage infiltration and polarization according to tumor stage and clinical outcome. Representative photomicrographs of H&E staining (**A**,**E**,**I**,**M**) and immunohistochemical staining for CD68 (**B**,**F**,**J**,**N**), CD11c (**C**,**G**,**K**,**O**), and CD163 (**D**,**H**,**L**,**P**). (**A**–**D**) pT1 case without recurrence or secondary lymph node metastasis. (**E**–**H**) pT1 case with recurrence or secondary lymph node metastasis. (**I**–**L**) pT2 case without recurrence or secondary lymph node metastasis. (**M**–**P**) pT2 case with recurrence or secondary lymph node metastasis. (**Q**–**S**) Comparison of the numbers of CD68-, CD11c-, and CD163-positive cells between pT1 and pT2 cases. No significant differences were observed in the numbers of CD68- or CD11c-positive cells between pT1 and pT2 cases, whereas the number of CD163-positive cells was significantly higher in pT2 than in pT1 cases (*p* < 0.01). (**T**,**U**) Comparison of the numbers of CD68-, CD11c-, and CD163-positive cells in pT1 and pT2 cases. In pT2 cases, CD163-positive cells were significantly more abundant than CD68-positive cells (*p* < 0.0001). (**V**,**W**) Quantification of the CD11c/CD68 and CD163/CD68 ratios. The CD163/CD68 ratio was significantly higher in pT2 than in pT1 cases (*p* < 0.01). (**X**) Comparison of the numbers of CD68-, CD11c-, and CD163-positive cells between cases with and without recurrence or secondary lymph node metastasis in the pT1 and pT2 groups. No significant differences were observed in any comparison. SLMN, secondary lymph node metastasis. Data are presented as the mean ± standard deviation (SD). ns, *p* > 0.05; ** *p* < 0.01; *** *p* < 0.001; **** *p* < 0.0001.

**Figure 4:**
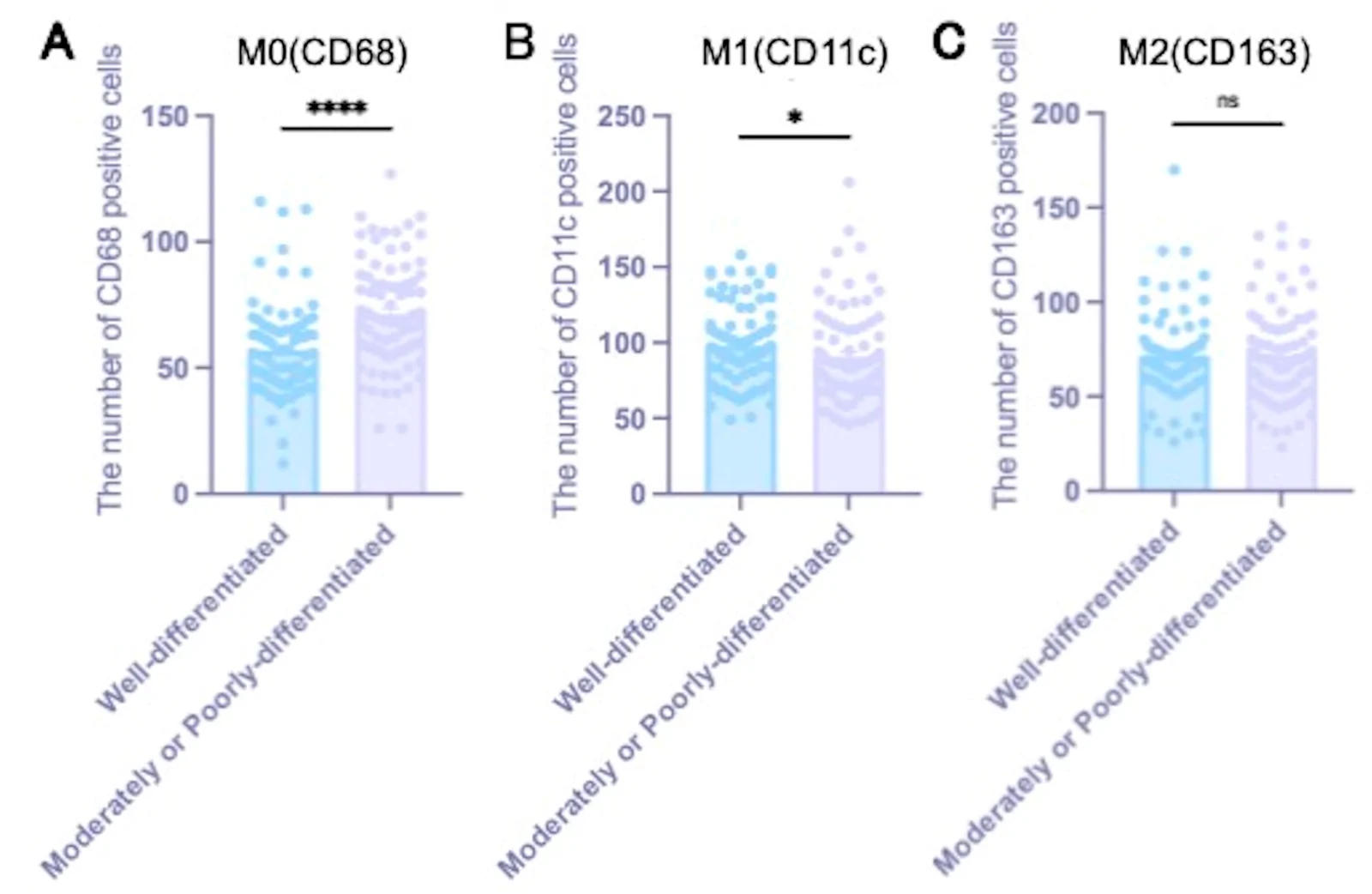

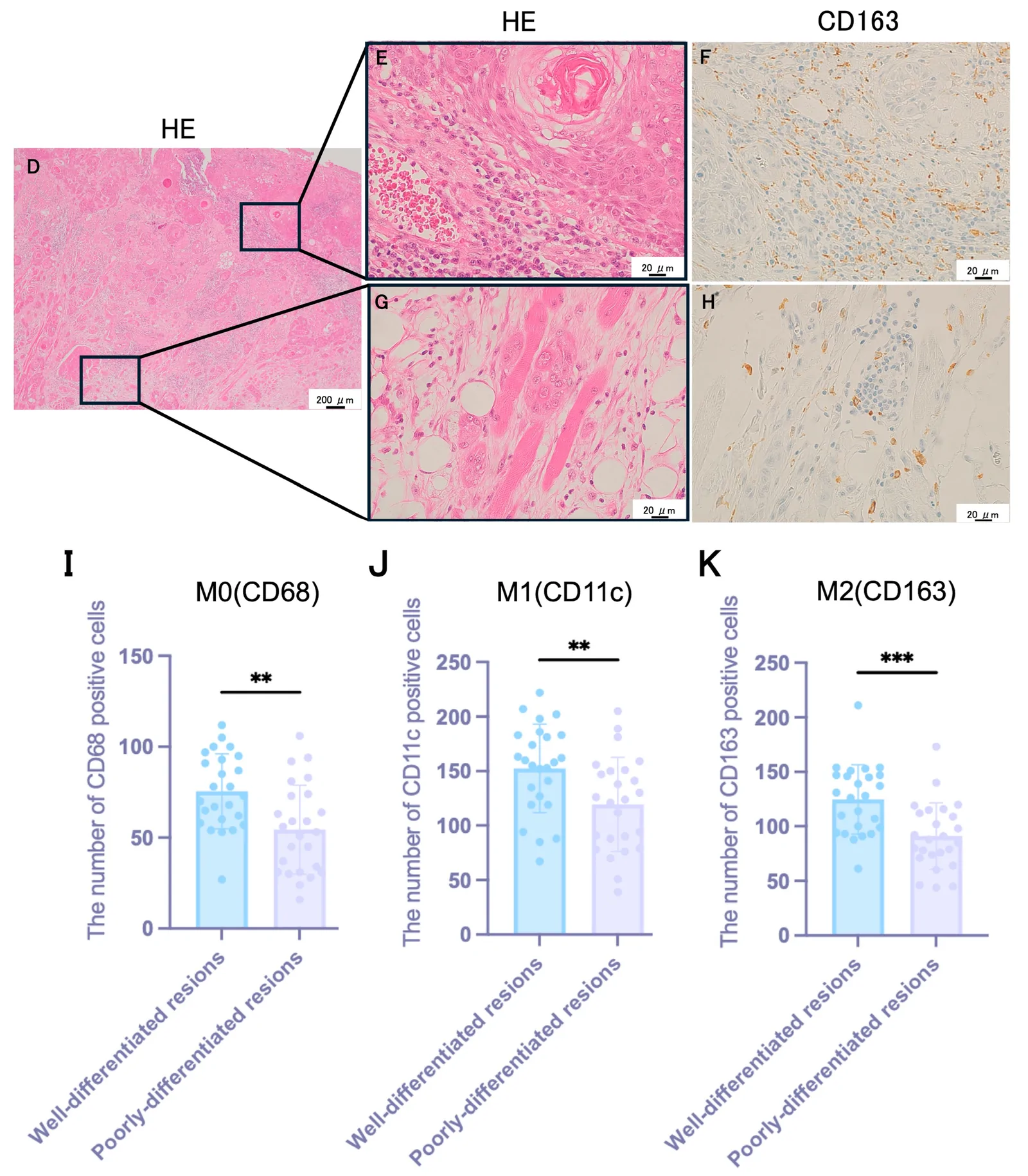
Regional differences in macrophage infiltration and polarization according to histological differentiation in human OSCC. (**A**–**C**) Quantification of CD68-, CD11c-, and CD163-positive cells according to the overall histological differentiation of OSCC. Compared with well differentiated SCC, moderately or poorly differentiated SCC exhibited a significantly higher number of CD68-positive cells (*p* < 0.0001), whereas well differentiated SCC contained significantly more CD11c-positive cells (*p* < 0.05). No significant difference was observed in the number of CD163-positive cells. (**D**–**H**) Representative images demonstrating intratumoral histological heterogeneity within the same tumor. (**E**,**F**) Well differentiated region. (**G**,**H**) Poorly differentiated region. CD163-positive cells were more abundant in the well differentiated region than in the poorly differentiated region. (**I**–**K**) Regional analysis of CD68-, CD11c-, and CD163-positive cells within the same tumors (n = 5). All three macrophage markers were significantly more abundant in well differentiated regions than in poorly differentiated regions (CD68, *p* < 0.01; CD11c, *p* < 0.01; CD163, *p* < 0.001). Data are presented as the mean ± SD. ns, *p* > 0.05; * *p* < 0.05; ** *p* < 0.01; *** *p* < 0.001; **** *p* < 0.0001.

**Figure 5:**
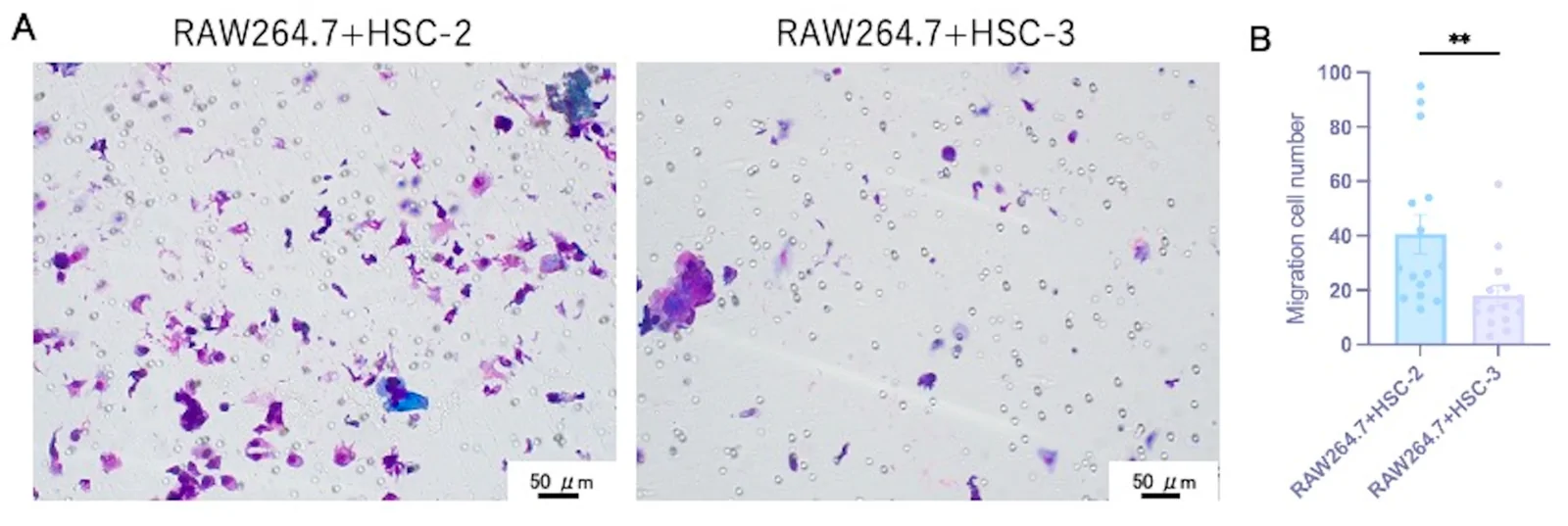

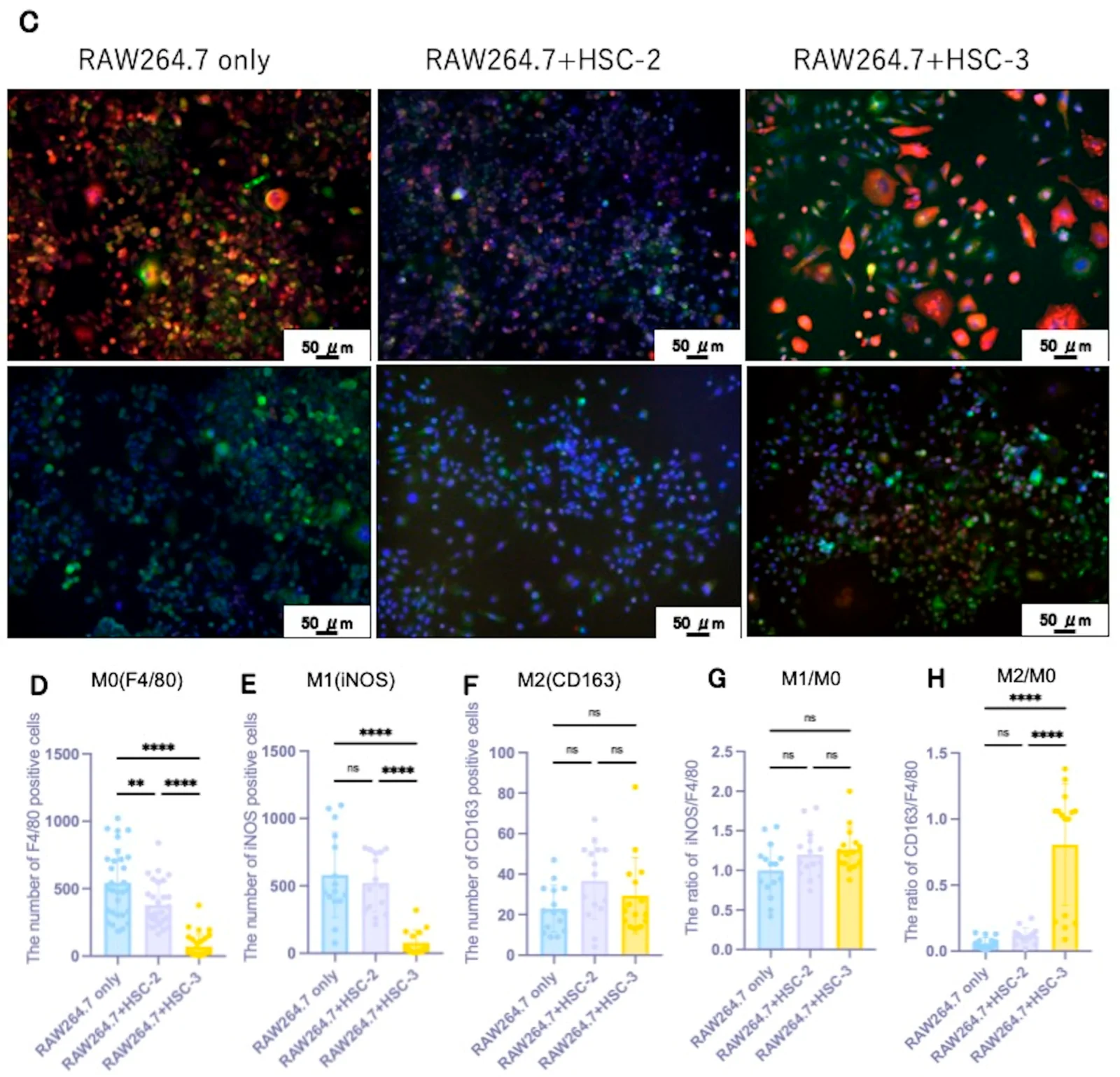
Macrophage migration and polarization in vitro. (**A**) Representative images of migrated RAW264.7 cells following co-culture with HSC-2 or HSC-3 cells, visualized by Giemsa staining. (**B**) Quantification of migrated RAW264.7 cells. Significantly more macrophages migrated toward HSC-2 cells than toward HSC-3 cells. (**C**) Effects of HSC-2 and HSC-3 cells on macrophage polarization in an indirect co-culture system. Left: RAW264.7 monoculture. Middle: Indirect co-culture of RAW264.7 and HSC-2 cells. Right: Indirect co-culture of RAW264.7 and HSC-3 cells. Upper panels: Double-immunofluorescence staining for F4/80 (green) and iNOS (red) with DAPI nuclear counterstaining (blue). Lower panels: Double-immunofluorescence staining for F4/80 (green) and CD163 (red) with DAPI nuclear counterstaining (blue). (**D**–**F**) Quantification of F4/80-, iNOS-, and CD163-positive cells in the RAW264.7 monoculture, RAW264.7 + HSC-2, and RAW264.7 + HSC-3 groups. The numbers of F4/80-positive cells differed significantly among the three groups. The number of iNOS-positive cells was significantly higher in the RAW264.7 + HSC-2 group than in the RAW264.7 + HSC-3 group (*p* < 0.0001). Although the number of CD163-positive cells showed a decreasing trend from the RAW264.7 + HSC-2 group to the RAW264.7 + HSC-3 group, the difference was not statistically significant. (**G**,**H**) Quantification of the iNOS/F4/80 and CD163/F4/80 ratios. No significant difference was observed in the iNOS/F4/80 ratio, whereas the CD163/F4/80 ratio was significantly higher in the RAW264.7 + HSC-3 group than in the RAW264.7 + HSC-2 group (*p* < 0.0001). n = 3. Data are presented as the mean ± standard deviation (SD). ns, *p* > 0.05; ** *p* < 0.01; **** *p* < 0.0001.

**Table 1:** Clinical findings of OED and early invasive SCC.

Factors		Low-Grade Dysplasia (n = 16)	High-Grade Dysplasia (n = 16)	Early Invasive SCC (n = 8)
Sex				
	Male	8	7	5
	Female	8	9	3
Age(year)		29~81 (median: 67.5)	31~84 (median: 62)	45~87 (median: 73)

**Table 2:** Clinical findings of OSCC cases.

Factors	T1 (n = 16)	T2 (n = 16)
Sex		
Male	12	10
Female	4	6
Age(year)	27~83 (median: 49.5)	28~79 (median: 63)
Tumor differentiation		
Well differentiated type	6	10
Moderately or Poorly differentiated type	10	6
Postoperative local recurrence	1	1
Postoperative lymph node metastasis	7	7

## Data Availability

The raw data that support conclusions of this study are available from the authors upon reasonable request. The data are not publicly available due to ethical and privacy restrictions related to the use of patient clinical and pathological information. The data supporting the findings of this study are available from the corresponding author upon reasonable request and with permission from the appropriate institutional authorities.
